# Loss-of-function mutations in *KEAP1* drive lung cancer progression via KEAP1/NRF2 pathway activation

**DOI:** 10.1186/s12964-020-00568-z

**Published:** 2020-06-23

**Authors:** Meiling Gong, Yan Li, Xiaoping Ye, Linlin Zhang, Zhifang Wang, Xiaowen Xu, Yejing Shen, Cuixia Zheng

**Affiliations:** 1 Department of Respiratory and Critical Care Medicine, Yangpu Hospital, Tongji Universtiy School Of Medicine, Shanghai, China; 2 Shanghai Jiaotong University School of Medicine, Shanghai, China; 3 Shanghai Ninth People’s Hospital, Shanghai Jiaotong University, Shanghai, China; 4 Shanghai University of Traditional Chinese Medicine, Shanghai, China

**Keywords:** KEAP1/NRF2, Somatic mutation, NRF2 inhibitor, Lung carcinoma, Targeted therapy

## Abstract

**Background and purpose:**

Targeted therapy and immunotherapy have led to dramatic change in the treatment of lung cancer, however, the overall 5-year survival rate of lung cancer patients is still suboptimal. It is important to exploit new potential of molecularly targeted therapies. High-frequency somatic mutations in *KEAP1/NRF2* (27.9%) have been identified in lung squamous cell carcinoma. In this research, we explored the role of *KEAP1* somatic mutations in the development of LSCC and whether a nuclear factor erythroid 2-related factor 2(NRF2) inhibitor be potential to target lung cancer carrying *KEAP1/NRF2* mutations.

**Methods:**

Lung cancer cell lines A549 and H460 with loss-of-function mutations in *KEAP1* stably transfected with wild-type (WT) *KEAP1* or somatic mutations in *KEAP1* were used to investigate the functions of somatic mutations in *KEAP1*. Flow cytometry, plate clone formation experiments, and scratch tests were used to examine reactive oxygen species, proliferation, and migration of these cell lines.

**Results:**

The expression of NRF2 and its target genes increased*,* and tumor cell proliferation, migration, and tumor growth were accelerated in A549 and H460 cells stably transfected with *KEAP1* mutants compared to control cells with a loss-of-function *KEAP1* mutation and stably transfected with WT *KEAP1* in both in vitro and in vivo studies. The proliferation of A549 cell line trasfected with the R320Q *KEAP1* mutant was inhibited more apparent than that of the A549 cell line trasfected with WT *KEAP1* after treatment with NRF2 inhibitor ML385.

**Conclusion:**

Somatic mutations of *KEAP1* identified from patients with LSCC likely promote tumorigenesis mediated by activation of the KEAP1/NRF2 antioxidant stress response pathway. NRF2 inhibition with ML385 could inhibit the proliferation of tumor cells with *KEAP1* mutation.

Video abstract

## Background

Lung cancer is a leading cause of cancer-related death, with a 5-year overall survival rate less than 15% [1], a significantly lower survival rate than that of most epithelial malignancies. Lung cancer is divided into small cell lung cancer and non-small cell lung cancer (NSCLC). The proportion of the NSCLC type is more than 85%, mainly including lung adenocarcinoma and lung squamous cell carcinoma [2, 3]. Lung squamous cell carcinoma (LSCC) accounts for approximately 30% of all lung cancers, with a extremely high mortality rate [4]. Because most lung cancer patients are already in the advanced stage of disease at the time of diagnosis, they have lost the opportunity for surgical treatment, and the prognosis of LSCC has not obviously improved, although the finding of high-frequency mutations in epidermal growth factor receptor (*EGFR*) kinase has led to a dramatic change in the treatment of patients with lung adenocarcinoma [5, 6], and recent data have indicated that targeting mutations in *BRAF, AKT1*, *ERBB2,* and *PIK3CA* as well as fusions that involve receptor tyrosine kinase genes *ALK*, *ROS1,* and *RET* may also be successful [7, 8]. Unfortunately, the activating mutations in *EGFR* and *ALK* fusions are limited in lung adenocarcinoma and are not present in LSCC [9], and targeted agents developed for these activating mutations are largely ineffective in LSCC.

Recent researches have accumulated approximately 29 possible pathogenic genes for LSCC and are widely accepted [10–12]. However, therapeutic drugs targeting these driver genes are lacking. Interestingly, a search of the TCGA database revealed that approximately 30% of LSCCs undergo recurrent mutations in *KEAP1* and *NFE2L2(also named as NRF2)* [11, 12]. In our previous study, we identified that *KEAP1* and *NRF2* mutations are recurrent in Chinese patients with LSCC, with a 5.8% frequency for *KEAP1* and a 27.9% frequency for *KEAP1/NRF2* mutations. However, mutations in *KEAP1/NRF2* in Chinese patients with lung adenocarcinoma are rarely found, which is consistent with reports from Takahashi T [13]. Interestingly, *KEAP1* and *NRF2* mutations show mutual exclusive in Chinese patients with LSCC [12]. *KEAP1* and *NRF2* are the two key genes that regulate the oxidative stress pathway. At physiological homeostasis, NRF2 is bound by the adapter protein KEAP1, which recruits the CUL3 ubiquitin ligase, leading to the proteasomal degradation of NRF2 [14]. Oxidative stress acts on KEAP1, causing its conformation change and dissociation from NRF2, thereby losing the ability to mediate NRF2 degradation [15, 16] and leading to NRF2 activation and subsequent antioxidative properties, which is important in maintaining physiological homeostasis. However, it has been reported that NRF2 activation involves in chemotherapy drugs inactivation through rapid metabolism of these drugs in cells, significantly reducing their anti-tumor efficacy [17–19]. More recently, the data have also shown that loss of function of *KEAP1* promotes *KRAS*-driven lung cancer and results in the dependence on glutaminolysis [20].

Therefore, we aimed to test whether mutations in *KEAP1*, identified in our previous study, accelerate the development of lung cancer, and whether a NRF2 inhibitor can be used as a targeted therapeutic drug in patients with lung cancer carrying *KEAP1/NRF2* mutations.

## Materials and methods

### Cell culture, reagents, and nude mice

The NCI-H1299,A549, H838, H460,H1299, 95D, and SPCA1 human lung cancer cell lines and HEK293T cells were obtained from American Type Culture Collection (Manassas, VA, USA). H1299, H838, H460, H292, 95D, and SPCA1 cells were maintained in RPMI 1640 medium (Gibco, Grand Island, NY, USA). A549 cells were cultured in F-12 K(Gibco) supplemented with 10% fetal bovine serum (Gibco) at 37 °C in a humidified atmosphere containing 5% CO2. Twelve 4–6-week-old male BALB/c nude mice were purchased and reared from the Shanghai Ninth People’s Hospital Central Laboratory Animal Law.

### Plasmids, site-directed mutagenesis, and stable transfection

Mutations were conducted using the QuikChange site-directed mutagenesis kit (Stratagene, La Jolla, CA, USA) and were validated by sequencing; the primer sequences for mutagenesis are shown in (Supplementary Table 1). A retrovirus-mediated infection system was used to construct A549 and H460 cells stably over-expressing 3FLAG-tagged KEAP1(WT or mutant). For PMSCV production, DNA encoding 3FLAG-tagged KEAP1 was inserted into the multi-cloning site of the pMSCV vector. Each PMSCV vector was co-transfected with gag-pol and VSVG using Lipofectamine 2000(Invitrogen,Waltham, MA, USA) in 293 T cells. The virus was collected 2 days later and was transfected into A549 and H460 cells. The infected cells were selected with 1 μg/mL (A549) or 0.5 μg/mL (H460) of puromycin for 3–4 weeks.

### Gene editing using CRISPR/Cas9 system

Target-specific guide RNA within NRF2 gene locus was designed on CRISPRDESIGN (http://crispr.mit.edu/). The following target sgRNA sequences were used in this study:sgRNA-F 5′-TGCCTGTAAGTCCTGGTCAT-3′, sgRNA-R 5′-TCTCTGGTGTGTTCTCACAT-3′. Igonucleotides for guide RNA were inserted into CRISPR Nuclease vector and then the vector was transfected into A549 cells using Lipofectamine 2000 (Invitrogen) according to the manufacturer’s instructions. Specific lentivirus transfecting method was the same as above. Finally, 3 homozygous knockout cell lines were selected (Supplementary Fig. 1a).

### Western blot and immunoprecipitation analyses

The antibodies used in our study were as follows: anti-FLAG M2 monoclonal (Sigma, St. Louis, MO, USA), anti-NRF2 (Abcam, Cambridge, UnitedKingdom), anti-β-actin (Cell Signaling,Danvers, MA, USA), anti-HO-1 (Cell Signaling), anti-LaminB1 (Cell Signaling), anti-Lamin A/C (Cell Signaling). The Nuclear and Cytoplasmic Protein Extraction kit was obtained from (Thermo Fisher Scientific, Waltham, MA, USA). For immunoprecipitation, Whole cell lysate (WCL) was used respectively as the negative control. Cell lysates were cleared by centrifugation and were incubated with FLAG resin (Sigma) before washing with lysis buffer, followed by overnight incubation at 4 °C. After washing three times by 1× phosphate-buffered saline (PBS), the precipitates were analyzed by immunoblotting.

### Real-time quantitative PCR

Total RNA was prepared from cells using Trizol reagent (Invitrogen), and tumors using Animal Total RNA Isolation kit (Sangon Biotech,Shanghai, China) and reverse transcription were performed using the PrimeScript RT reagent kit with gDNA Eraser (Takara Bio,Shiga, Japan). The sequences for each primer are listed in Supplementary Table 2.

### ROS measurement

After the cells were washed with PBS twice, the cells were incubated with 1 μM CM-H2DCF-DA (Nanjing Jiancheng, Nanjing, China) in culture conditions for 30 min in the dark and then were trypsinized and collected. The ROS levels were examined by flow cytometry.

### Colony-formation assay

Exponentially growing cells were counted,diluted, and seeded in triplicate at 800–1000 cells/well in 6-well plates. To assess clonogenic survival following drug exposure, the cell cultures were incubated in complete growth medium at 37 °C for 11–14 days. Colonies were fixed with precooled methanol and then were stained with 0.5% (w/v) crystal violet for 30 min at room temperature, followed by washing with PBS, photographing and counting. Only colonies with more than 50 cells were counted [21].

### Wound-healing assay

Cell motility was determined by measuring the movement of cells close to an artificial wound. Cells were wounded with a 200-μL pipette tip, washed with PBS, and incubated in F12-K or RPMI 1640 medium without FBS. The distances removed by cells were monitored by microscopy at the indicated time points. The scratched area was analyzed using ImageJ.

### Tumor xenograft model

Twelve BALB/c nude mice (4–6 weeks old, male) were randomly assigned to two groups (WT or mutant). We infected A549-*KEAP1*-WT or A549-*KEAP1*-R320W cells (1 × 10^8^) subcutaneously into the right flank of BALB/c nude mice and measured the tumor dimensions by calipers every 3–4 days. The tumor volumes were calculated using the formulalength [(mm) × width (mm)^2^]/2 [22]. All experimental protocols conducted on mice were performed in accordance with the National Institutes of Health (NIH) guidelines and were approved by the Shanghai Jiaotong University Animal Care and Use Committee.

### Statistical analysis

All experiments were performed in quadruplicate and were repeated at least three times with similar results unless otherwise indicated. All statistical analyses were performed using unpaired two-tailed Student’s t-test and the mean ± standard error of the mean. A *P*-value of 0.05 or less was considered statistically significant. These analyses were performed using SPSS 13.0 software (SPSS Inc., Chicago, IL, USA) or GraphpadPrism 7 (GraphpadSoftware, San Diego, CA, USA).

## Results

### The KEAP1/NRF2 pathway was activated in lung cancer cell lines with *KEAP1* mutations

**Fig. 1:**
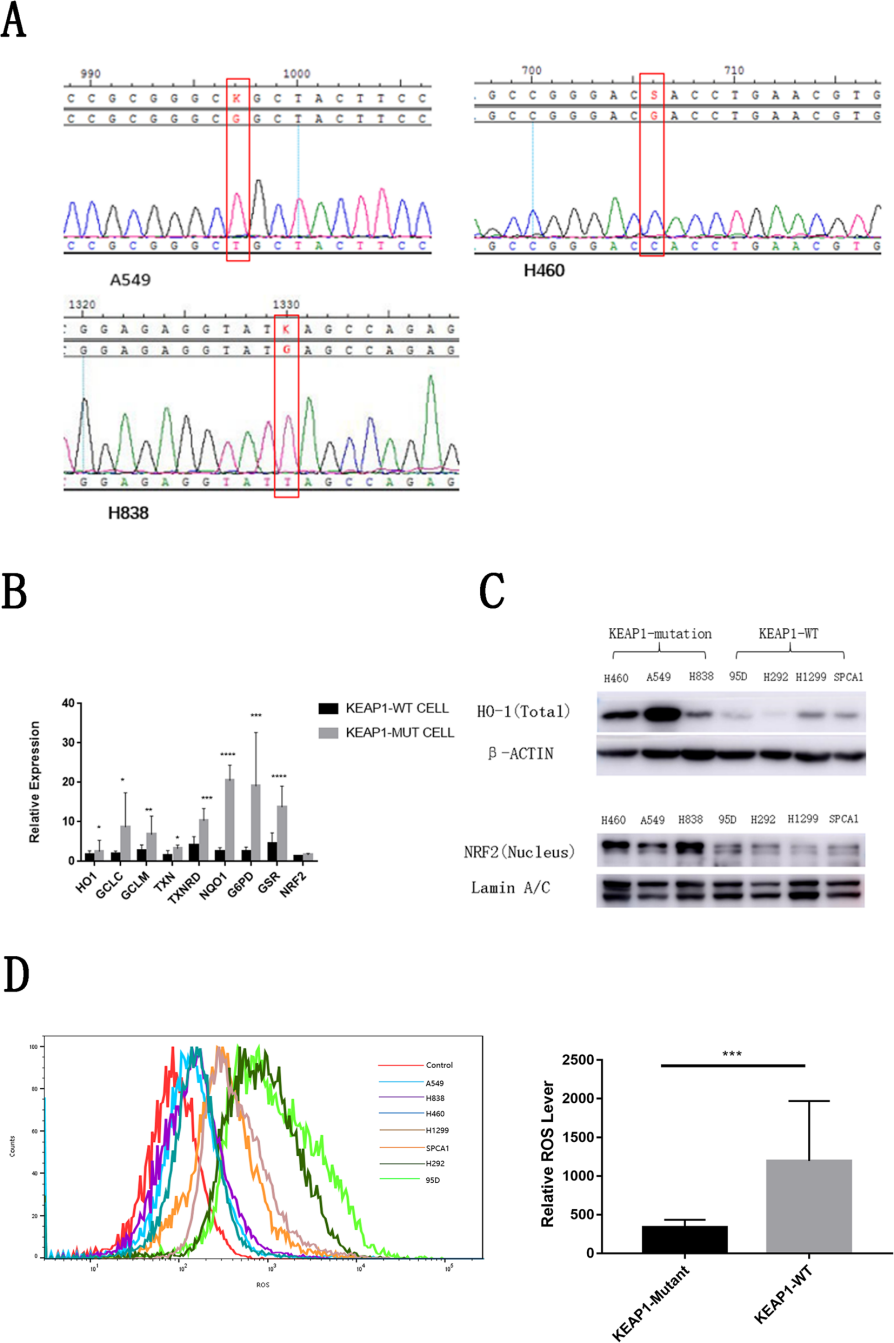
The KEAP1/NRF2 pathway was activated in lung cancer cell lines with loss-of-function mutations in *KEAP1*. **a** The mutations of *KEAP1* in A549, H460, and H838 cells were detected by Sanger sequencing. The red rectangle indicates the mutation sites of *KEAP1*. **b** Compared with lung cancer cells with neither *KEAP1* nor *NRF2* mutation, the expression levels of *NRF2* target genes were significantly increased in lung cancer cells with *KEAP1* mutation. **c** The protein expression levels of NRF2 and its target protein HO-1 were increased in lung cancer cells with *KEAP1* mutation compared with those in lung cancer cells with neither *KEAP1* nor *NRF2* mutation. **d***KEAP1* mutation suppressed ROS production in lung cancer cells. The fluorescence intensity in cells was detected by flow cytometry. Quantitative analysis of the mean fluorescence intensity using unpaired two-tailed Student’s t-test. The results are expressed as mean ± standard error of the mean. * *P* < 0.05, ** *P* < 0.01, *** *P* < 0.001, and **** *P* < 0.0001

To explore the effect of *KEAP1* loss on activation of the KEAP1/NRF2 pathway, we collected three NSCLC cell lines with *KEAP1* mutations (A549, NCI-H460, and NCI-H838) and four NSCLC cell lines without *KEAP1/NRF2* mutations (NCI-H1299, NCI-H292, 95D, and SPCA1). In the present study, we have found the frequency of *KEAP1/NRF2* mutation was higher in human LSCC than that in LUAD patients, thus we hope find the LSCC cell lines with *KEAP1* mutation to investigate the effect of *KEAP1* mutation on the function of *KEAP1/NRF2* pathaway. However, after serching the literatures and cell line database, we found none LSCC cell lines carried *KEAP1/NRF2* mutation. We further collected all available 7 lung cancer cell lines and found three of them carried the *KEAP1* mutation (A549, NCI-H460, NCI-H838), thus, in the present study, all the seven lung cancer cell lines were used to study the influence of *KEAP1* mutation on the function of *KEAP1/NRF2* pathway. As expected, Sanger sequencing revealed that the A549, H460, and H838 cell lines carried homozygous point mutations at D236H, G333C, and E444* in *KEAP1*, respectively; however, H1299, NCI-H292, 95D, and SPCA1 cell lines did not carry mutations in neither *KEAP1* nor *NRF2***(**Fig. 1a). Next, we found that the mRNA expression of downstream target genes of the KEAP1/NRF2 pathway, such as *GCLC*, *GCLM*, *TXN*, *TXNRD*, *HO1*, *NQO1*, *GSR,* and *G6PD*, which encode detoxifying enzymes and antioxidant proteins, were significantly higher in *KEAP1* mutant cell lines than in wild-type (WT) lung cancer cells by real-time polymerase chain reaction (PCR), but the mRNA expression of *NRF2* showed no significant difference between lung cancer cell lines with and without *KEAP1* mutation **(**Fig. 1b**)**. Given that NRF2 protein translocates into the nucleus to activate the transcription of downstream target genesin the KEAP1/NRF2 pathway, we further examined the protein expression of nuclear NRF2 and the downstream target HO-1 in these tumor cells by western blot analysis. As expected, function loss of *KEAP1* significantly increased nuclear NRF2 levels and HO-1 levels incytoplasm (Fig. 1c). Thus, *KEAP1* loss decreased the degradation of NRF2 to activate the KEAP1/NRF2 pathway.

Activation of the KEAP1/NRF2 pathway decreases intracellular reactive oxygen species (ROS) levels through upregulating the expression of detoxifying enzymes and antioxidant genes. Thus, we checked whether *KEAP1* loss could decrease ROS production in lung cancer cell lines. As shown in Fig. 1d, ROS levels were significantly decreased in lung cancer cells with *KEAP1* mutation. Collectively, these findings demonstrate that *KEAP1* loss can enhance the ability of lung cancer cells to resist oxidative stress.

### Mutations in *KEAP1* identified in Chinese patients with lung cancer promoted tumorigenesis via activation of the KEAP1/NRF2 pathway in lung cancer cells

In our previous study, we identified five nonsynonymous mutations in *KEAP1* from five patients with LSCC. However, the role of these *KEAP1* mutations in tumorigenesis is unclear. The function these five nonsynonymous mutations in *KEAP1* were predicted by *Polyhen2_HDIV* and *SIFT*. The results showed that these five nonsynonymous mutations in *KEAP1* were harmful, affecting protein function (Table 1).

**Fig. 2:**
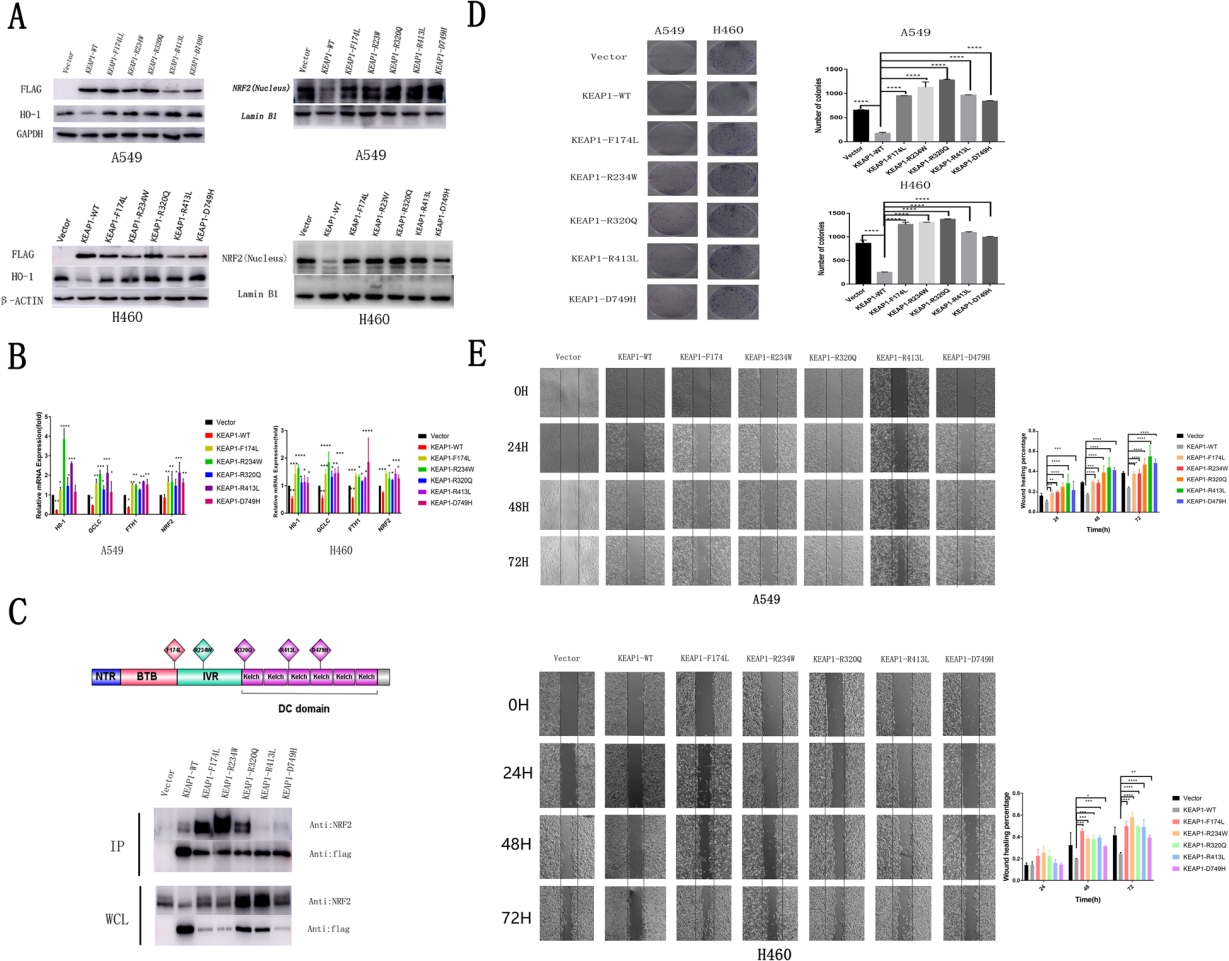
*KEAP1* mutation promoted the activity of lung cancer cell lines in vitro. **a** Compared with A549 or H460 lung cancer cell lines transfected with empty vector, expression levels of nuclear NRF2 and its target protein HO-1 were significantly decreased in A549 or H460 cells transfected with wild-type (WT) *KEAP1* by western blot analysis. The levels of nuclear NRF2 and its target gene HO-1 showed no significant difference after A549 or H460 cells were transfected with the five *KEAP1* mutants. **b** The mRNA expression levels of *NRF2* and its target genes were significantly decreased after the cell lines were stably transfected with WT *KEAP1*. However, the mRNA levels of *NRF2* and its target genes *HO-1*, *GCLC,* and *FTH1* were significantly increased in A549 or H460 lung cancer cell lines stably transfected with the five *KEAP1* mutants. **c** Position of the mutation in the KEAP1 protein. Some mutants (R174L, R234W, and R320Q) enhanced NRF2 binding, while others (R413L and D749H) weakened NRF2 binding. Flag-tagged KEAP1 was co-expressed in A549 cells. Whole cell lysate (WCL) was used respectively as the negative control. Immunoprecipitation experiments were performedusing anti-flag M2 beads, and immunoblot analysis was performed using anti-flag and anti-NRF2 antibodies. **d** Colony-formation assay showed that the proliferation of A549/H460 lung cancer cell lines stably transfected with WT *KEAP1* was significantly decreased, while that of cells transfected with mutant *KEAP1* was significant increased. **e** The scratch wound-healing assay showed that the migration of A549/H460 cells stably transfected with mutant *KEAP1* was faster at 0 h, 24 h, 48 h, and 72 h than that of A549/H460 cells transfected with WT *KEAP1*. Scratch area quantitative analysis by Image J software. Each assay was repeated at least three times. Mean ± standard error of the mean (SEM) are reported (* *P* < 0.05; **, *P* < 0.01; ***, *P* < 0.001)

To verify whether these five somatic mutations in *KEAP1* influence the function of *KEAP1*, WT and five *KEAP1* mutants were stably transfected with retroviral vectors into A549/H460 lung cancer cell lines that carry loss-of-function mutations in *KEAP1*. As expected, nuclear NRF2 protein levels and expression of the NRF2 target gene *HO-1* were significantly decreased after A549 or H460 lung cancer cell lines were stably transfected with WT *KEAP1* (Fig. 2a). However, the levels of nuclear NRF2 protein and expression of NRF2 target gene *HO-1* showed no significant difference after A549 or H460 lung cancer cell lines were stably transfected with these five *KEAP1* mutants (Fig. 2a). These lung cancer cell lines that carry loss-of-function mutations in *KEAP1*, were losing the ability to mediate NRF2 degradation. Activated NRF2 will be degradated when WT *KEAP1* overexpression.

Compared with A549 or H460 lung cancer cell lines stably transfected with empty vector*,* mRNA levels of *NRF2* and its target genes *HO-1*, *GCLC,* and *FTH1* were significantly decreased after the cell lines were stably transfected with WT *KEAP1* (Fig. 2b). However, mRNA levels of *NRF2* and its target genes *HO-1*, *GCLC,* and *FTH1* were significantly increased in the A549 or H460 lung cancer cell lines stably transfected with these five *KEAP1* mutants (Fig. 2b). Together, these data suggested that these five nonsynonymous mutations in *KEAP1,* derived from Chinese patients with LSCC, were loss-of-function mutations that upregulated the expression of detoxifying enzymes and antioxidant genes.

*KEAP1* is located at 19p13.2 and its protein has three major domains: an N-terminal broad complex, tramtrack, and the bric-a-brac (BTB) domain; a central intervening region (IVR); and a series of six C-terminal Kelch repeats [23]. The Kelch repeats of KEAP1 bind the NEH2 domain of NRF2 [24], whereas the IVR and BTB domains are required for the redox-sensitive regulation of NRF2 through a series of reactive cysteines present throughout this region [14, 25]. In our present study, we found somatic mutations at R320Q, R413L, and D479H in the Kelch repeat domains of KEAP1, a somatic mutation at R234W in the IVR domain, and a somatic mutation at F174L in the BTB domain of KEAP1 (Fig. 2c). The binding of KEAP1 mutants to NRF2 was detected in coimmunoprecipitation experiments. Interestingly, mutants at R413L and D479H in the Kelch repeat domain of *KEAP1* did not bind to NRF2 (Fig. 2c). However, compared with WT KEAP1, binding of the mutants at F174L in the BTB domain and at R234W in the IVR domain of NRF2 was significantly increased (Fig. 2c). Unexpectedly, binding of NRF2 to the *KEAP1* mutants at R320Q in the Kelch repeat domain was not affected (Fig. 2c).

To uncover whether these five somatic mutations in KEAP1 influence the biological behavior of lung cancer cells, cell proliferation and migration were detected by colony-formation and scratch experiments. Compared with A549/H460 lung cancer cell lines stably transfected with retroviral empty vector, the colony formation and migration of A549/H460 lung cancer cell lines stably transfected with WT KEAP1 were significantly decreased (Fig. 2d, e). However, after being stably transfected with KEAP1 mutants, the colony formation and migration of A549/H460 lung cancer cell lines significantly increased (Fig. 2d, e). To further validate that their mechanism were really passed through the NRF2 pathway, we knockdown NRF2 using double sgRNAs and detected the expression of well established downstream genes of NRF2. As shown in (supplementary Fig. 1b, c), the expression of NRF2 target gene HO-1protein level was significantly decreased and the mRNA level of HMOX1,HO-1, GCLC, NQO1, FTH1, NRF2 when NRF2 was knockdown. As expected (supplementary Fig. 1d, e), the reduced number of colonies and migration in cells knockouted with NRF2.These data suggest that the newly found somatic mutations in KEAP1 promote tumor cell activity through activating NRF2 antioxidant stress signaling pathways.

### The somatic mutation at R320Q in *KEAP1* accelerated tumor growth in vivo

**Fig. 3:**
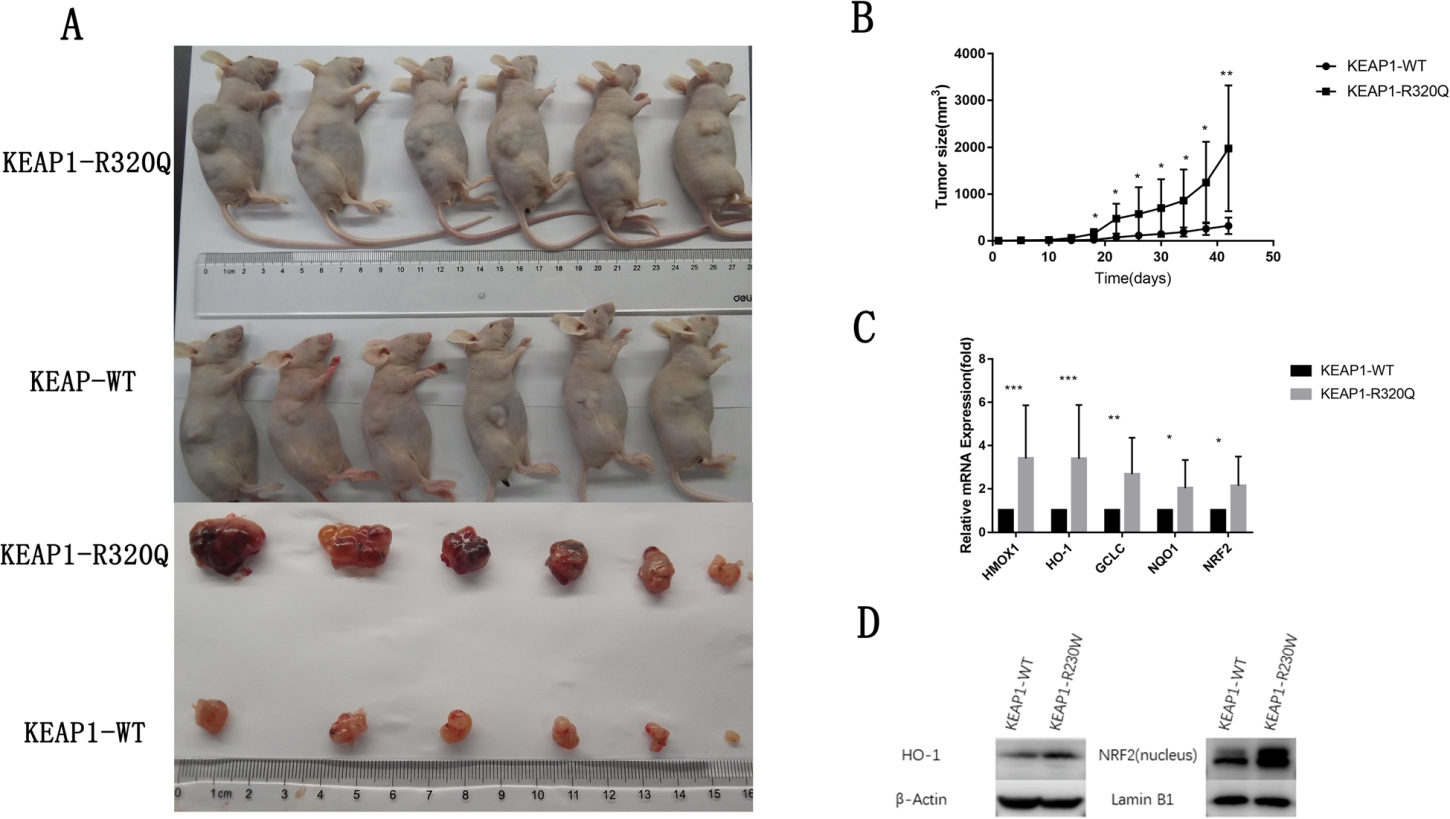
The somatic mutation R320Q in *KEAP1* accelerates tumor growth in vivo. **a** Tumor volumesof nude mice subcutaneously injected with A549 cells stably transfected with the *KEAP1* mutant (R320Q) were significantly larger than those injected with A549 cells transfected with wild-type (WT) *KEAP1*. Twelve 4–6-week-old male BALB/c nude mice were separated into two groups and injected with A549 cells transfected with WT *KEAP1* or mutant *KEAP1* (R320Q). Solid tumors were peeled from mouse subcutaneous tissues 7 weeks after injection. **b** Tumor growth was significantly faster in nude mice subcutaneously injected with A549 cells stably transfected with mutant *KEAP1* (R320Q) than those with A549 cell transfected with WT *KEAP1*. **c** The expression of *NRF2* and its target genes in xenograft tumors from A549 cells stably transfected with mutant *KEAP1* (R320Q) were higher than that from A549 cells transfected with WT *KEAP1*. Total RNA of xenograft tumors was extracted, and the indicated mRNA levels were determined by real-time PCR. **d** The nuclear protein levels of NRF2 and its downstream target protein HO-1 were increased in *KEAP1*-mutant (R320Q) xenograft tumors. Mean ± SEM are reported (* *P* < 0.05, ** *P* < 0.01, *** *P* < 0.001)

Due to we have considered that R320Q mutant has a considerable effection to KEAP1’S function, the ability of the antioxidative stress or proliferation and migration was significantly increased. Therefore, we choosed the R320Q mutant for our next experiments. To further examine the effect of the somatic mutations in *KEAP1* on the growth of lung cancer cell lines in vivo, the A549 cell lines stably transfected with WT *KEAP1* or the R320Q mutant of *KEAP1* were grafted subcutaneously into 4 to 5-week-old nude mice. After the cancer cell lines were grafted subcutaneously into nude mice, tumor sizes were measured using Vernier caliperseach day for 4 days. After 45 days of subcutaneous engraftment, tumors were peeled from the subcutis of the nude mice. The tumor sizes of the A549 cell line stably transfected with the R320Q *KEAP1*mutant were significantly larger than those of the A549 cell line stably transfected with WT *KEAP1***(**Fig. 3a). Additionally, the tumor growth of the A549 cell line stably transfected with the R320Q *KEAP1* mutant was strongly accelerated compared with that of the A549 cell line stably transfected with WT *KEAP1*, as measured by the change in tumor volume **(**Fig. 3b**).** Consistent with the in vitro results, the *KEAP1* mutant showed significantly accelerated tumor growth in vivo. These results indicate that *KEAP1* likely is a novel tumor driver gene for LSCC.

Next, we examined the expression of oxidative stress-related genes in the grafted tumor tissues from the nude mice. Compared with the expression of NRF2 in the nucleus and its target protein HO-1 in the cytoplasm in tumor tissues from the A549 cell line transfected with WT *KEAP1,* the expression levels of NRF2 and its target protein HO-1 were significantly increased in the tumor tissues from the A549 cell line transfected with the R320Q *KEAP1* mutant **(**Fig. 3d**).** The mRNA levels of *NRF2* and its target genes *HMOX1*, *HO-1*, *GCLC,* and *NQO1* in the grafted tumor tissues from the A549 cell line transfected with the R320Q *KEAP1* mutant were remarkably increased compared with that in grafted tumor tissues from the A549 cell line transfected with WT *KEAP1*(Fig. 3c**)**.

### The NRF2 inhibitor ML385 inhibited proliferation of lung cancer cells carrying *KEAP1* mutations

**Fig. 4:**
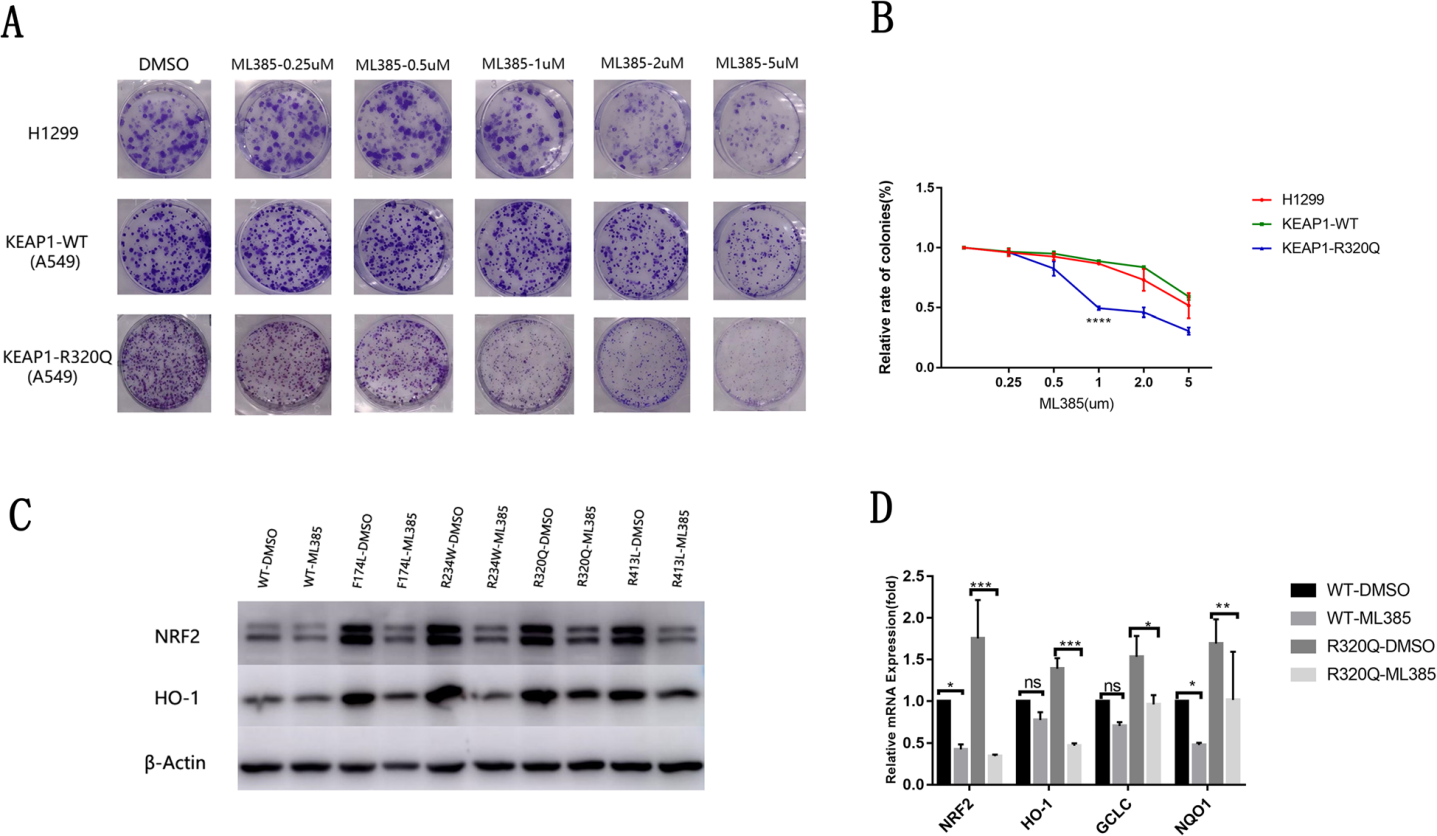
NRF2 inhibitor ML385 effectively inhibited proliferation of lung cancer cells carrying *KEAP1* mutations. **a** The number of formed colonies was decreased in the three groups as the dose of ML385 increased. When the lung cancer cell lines were treated with ML385 at a low dose (0.5 or 1.0 μM/L) the proliferation of A549 lung cancer cells transfected with the R320Q *KEAP1* mutant was more significantly inhibited than that of A549 cells transfected with wild-type (WT)*KEAP1* or that of H1299 lung cancer cells, which carried neither *KEAP1* nor *NRF2* mutation. Three groups of cells were treated with different doses of ML385 for 72 h. At the end of treatment, the medium was added and cells were further incubated for 8–10 days and stained with crystal violet. **b** The number of formed coloniesrapidly deceased in A549 cells transfected with the R320Q *KEAP1* mutant after treatment with ML385 compared with that in A549 cells transfected with WT *KEAP1* or H1299 lung cancer cells. Clonal formation rate = effective clones/plating cell numbers × 100% (*P < 0.05). **c** Protein levels of NRF2 and HO-1 were decreased in *KEAP1* mutant cells after drug intervention. Total protein was extracted after 3 days of treatment with 1 μM ML385. **d** The mRNA levels of NRF2 and HO-1 were decreased in *KEAP1* mutant cells. Total RNA was extracted after 3 days of treatment with 1 μMML385. Mean ± SEM are reported (* *P* < 0.05, ** *P* < 0.01, *** *P* < 0.001)

Increased cellular oxidative stress levels by small-molecule compounds to enhance cytotoxicity have been identified as a viable cancer treatment strategy [26]. High levels of ROS not only inhibit cancer cell proliferation but also trigger apoptosis. A subset of NRF2 inhibitors has been reported to inhibit the proliferation of cancer cells by down-regulating the expression of *NRF2*, resulting in elevated levels of intracellular ROS and increased cytotoxicity. However, it is unknown whether NRF2 inhibitors have different effects on these LSCCs with or without *KEAP1* somatic mutations. Thus, we selected an effective NRF2 inhibitor, ML385, which specifically and directly interacts with NRF2 protein, blocks *NRF2* transcriptional activity, and enhances the efficacy of carboplatin and other chemotherapeutic drugs in lung cancer cells [27]. The lung cancer cell line A549 transfected with the R320Q *KEAP1* mutant (*KEAP1-*R320Q mutant) or with WT *KEAP1* (*KEAP1-WT*) and H1299 lung cancer cells, which carry both WT *KEAP1* and *NRF2*, were selected and treated with ML385. The number of formed colonies was decreased in all three groups in a dose-dependent manner with increased ML385 treatment **(**Fig. 4a). Interestingly, when the lung cancer cell lines were treated with ML385 at a low dose, such as 0.25 or 0.5 μM/L, the proliferation of theA549 lung cancer cell line transfected with the R320Q *KEAP1* mutant showed more significant inhibition than that of A549 cells transfected with WT *KEAP1* or that of H1299 lung cancer cells **(**Fig. 4a, b). The proliferation of lung cancer cell lines showed no significant difference between A549 cells transfected with WT *KEAP1* and H1299 lung cancer cells without *KEAP1* and *NFR2* mutation **(**Fig. 4a, b). These results suggest that lung cancer cell lines with *KEAP1* mutations may have higher sensitivity to ML385 treatment.

To further detect whether the effect of ML385 on lung cancer cell proliferation is mediated by inhibiting the KEAP1/NRF2 pathway, the expression of *NRF2* and its target genes *HO-1, GCLC* and *NQO1* in lung cancer cell lines treated with ML385 were investigated by western blot and real-time PCR. As shown in Fig. 4c, the expression levels of HO-1 and NRF2 protein in A549 lung cancer cells transfected with F174L, R234W, R320Q, and R413L *KEAP1* mutants were significantly inhibited by ML385. However, the expression levels of HO-1 and NRF2 protein showed no significant difference between A549 transfected with WT *KEAP1* treated with and without ML385 (Fig. 4c). Although mRNA expression levels of *NRF2* and its target gene *NQO1* in A549 cells transfected with WT *KEAP1* were significantly inhibited by ML385, the mRNA expression levels of the *NRF2* target genes *GCLC* and *HO-1* were not influenced by ML385 (Fig. 4d). Notably, the mRNA expression levels of *NRF2* and its target genes *HO-1*, *GCLC,* and *NQO1* in A549 cells transfected with F174L, R234W, R320Q, and R413L *KEAP1* mutants were dramatically decreased after treatment with ML385 (Fig. 4d).

## Discussion

Recently, high-frequency somatic mutations of *KEAP1* and *NRF2* in the oxidative stress response pathway have been identified in patients with NSCLC by large-scale genomic studies [28]. Previous studies have revealed that *NRF2* is involved in cancer development, especially lung cancer [29–32]. In addition, recent evidence suggests that in mouse models of lung cancers, activated Nrf2 inhibits the Fbxo22-dependent degradation of Bach1 via induction of Ho-1 expression, and high levels of Bach1 promoting metastasis [33]. The loss-of-function mutation of *KEAP1* promoted the tumorigenesis of *Kras*- and *Pten*-driven lung cancer cell lines in mice [20, 34]. Moreover, the KEAP1/NRF2 pathway synergized with *TP53* deletion mutation could induce LSCC and radiation resistance [22]. However, no evidence that *KEAP1* or *NRF2* mutations identified in lung cancer patients are involved in tumorgenesis has been reported. In the present study, we found that the ability of the antioxidative stress response mediated by activation of the KEAP1/NRF2 pathway is higher in lung cancer cell lines with *KEAP1* mutation (A549, NCI-H460, NCI-H838) than in lung cancer cell lines without *KEAP1* mutation (NCI-H1299, NCI-H292, 95D, and SPCA1). Interestingly, compared with the lung cancer cell lines A549 and H460, which carry *KEAP1* mutation, after stable transfection with WT *KEAP1*, the mRNA levels of *NRF2* and its target genes are significantly increased in A549 and H460 lung cancer cell lines transfected with *KEAP1* mutants. Moreover, colony formation and migration were increased in A549 and H460 lung cancer cell lines transfected with *KEAP1* mutants*.* Similarly, the grafted subcutaneous tumor sizes in nude mice were significantly larger in A549 cells transfected with the R320Q *KEAP1* mutant than those inA549 cells transfected with WT *KEAP1*. These data suggest that the somatic mutations of *KEAP1* identified in Chinese patients with LSCC likely promote the development of lung cancer through activation of the antioxidative stress response in the KEAP1/NRF2 pathway.

Although genomic analysis identified some high-frequency gene mutations from LSCC, such as *TP53*, *PI3KCA*, and *SOX2*, no clear operationable targets for the treatment of LSCC have been found thus far [11, 12, 35]. Practically, molecular targeted therapy improves the survival of patients with lung adenocarcinoma, but no effective targeted drugs have been identifiedin clinical trials for LSCC [36]. Convenient treatment of LSCC remains to be platinum-based chemotherapy, added with immune checkpoint inhibitors that emerged recently and has brought certain benefits for the treatment of LSCC [37]. Additionally, cancer cells acquire novel nutrient dependencies to support oncogenic growth by changing metabolic pathways, Sarah E first points that dietary restriction or enzymatic depletion of asparagine can lead to suppression of Keap1 mutant tumor growth [38]. However, the overall effects of therapy for LSCC remain grim, revealing the immediate need for an effective treatment. In the present study, we found that the loss-of-function mutation in*KEAP1* promotes the development of lung cancer mediated by activation of the KEAP1/NRF2 pathway. Thus, it is tempting to presume that inhibitors of the KEAP1/NRF2 pathway will likely treat Lung cancer patients carrying *KEAP1/NRF2* mutations. To date, some inhibitors of the KEAP1/NRF2 pathway have been identified, such as NRF2 inhibitors that inhibit this pathway: ML385 [27], Brucea chinensis [39], clonazepa propionate [40], luteolin, all-trans retinoic acid, and flavonoid molecular compounds [41, 42]. The NRF2 inhibitor ML385 blocks activation of the pathway by inhibiting *NRF2* expression. We further explored the effect of ML385 on the development of lung cancer cell lines with or without *KEAP1* mutations. Compared with A549 lung cancer cell lines trasfected with WT *KEAP1*, proliferation of the A549 lung cancer cell line trasfected with the R320Q *KEAP1* mutant was dramatically inhibited by ML385. These preliminary datas suggest that ML385 inhibits the proliferation of lung cancer cells with *KEAP1* mutations by blocking the KEAP1/NRF2 antioxidant stress response pathway. It will provide a new option for therapy targeted to the KEAP1/NRF2 pathway in patients with LSCC carrying the *KEAP1* mutation.

## Conclusion

In summary, high-frequency mutation in *KEAP1* has been identified in Chinese patients with LSCC. The somatic nonsynonymous mutations in *KEAP1* derived from patients with lung cancer likely promote tumorigenesis via activation of the KEAP1/NRF2 antioxidant stress response pathway. Notably, NRF2 inhibitor ML385 may inhibit the proliferation of tumor cells with *KEAP1* mutation by enhancing the oxidative stress level of lung cancer cells.

## Supplementary information

**Additional file 1: Supplementary Fig. 1**. (A). Gene editing methods in A549 lung cancer cell lines with NRF2 homozygous knockout (The yellow part represents target sequences; the dotted line represents the base sequence of the knockout) (B). Compared with A549, expression NRF2 and its target protein HO-1 were significantly decreased in A549 with NRF2 knockout by western blot analysis. (C). The mRNA expression levels of *NRF2* and its target genes were significantly decreased after the cell lines were knockout NRF2.(D) Colony-formation assay showed that the proliferation of A549 lung cancer cell lines depleted with NRF2 was significantly decreased.(E) The scratch wound-healing assay showed that the migration of A549 lung cancer cell lines depleted with NRF2 was slower at 0 h,24 h, and 48 h than that of A549 cell lines. Mean ± standard error of the mean (SEM) are reported (* *P* < 0.05; **, *P* < 0.01; ***, *P* < 0.001).

## Data Availability

Not applicable.

## Abbreviations

NRF2/ *NFE2L2*: A nuclear factor erythroid 2-related factor 2
NSCLC: Non-smallcell lung cancer
LSCC: Lung squamous cell carcinoma
EGFR: Epidermal growth factor receptor
WT: Wild-type
*Polyhen2_HDIV*: http://genetics.bwh.harvard.edu/pph2/
*SIFT*: http://sift.jcvi.org/
ROS: Reactive oxygen species
