# Supplementary figures and images for: Loss-of-function mutations in KEAP1 drive lung cancer progression via KEAP1/NRF2 pathway activation
Source: Cell Commun Signal. 2020 Jun 23;18:98. doi: 10.1186/s12964-020-00568-z (PMC7310414; doi:10.1186/s12964-020-00568-z)

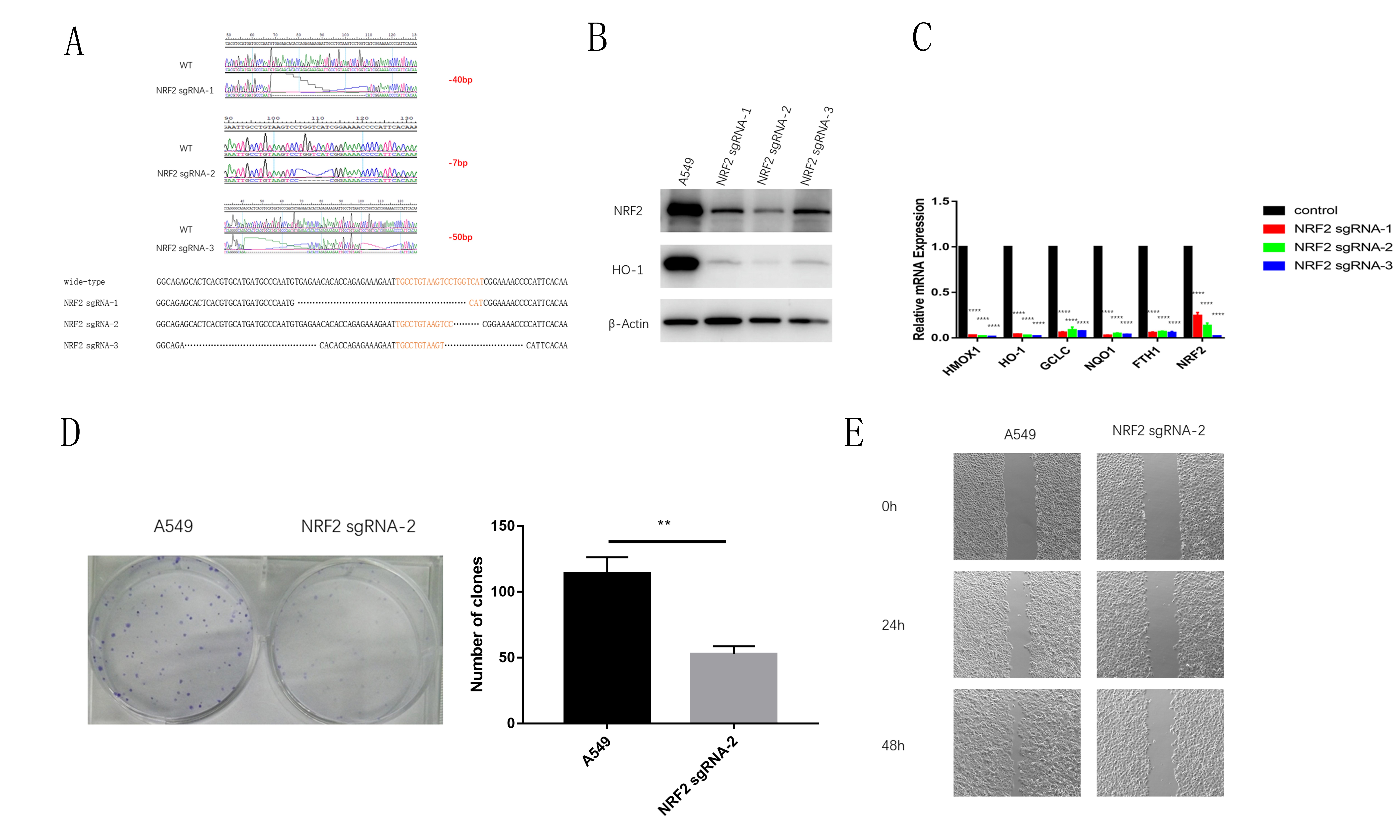

Supplement: Supplementary file 2 — Additional file 1: Supplementary Fig. 1. (A). Gene editing methods in A549 lung cancer cell lines with NRF2 homozygous knockout (The yellow part represents target sequences; the dotted line represents the base sequence of the knockout) (B). Compared with A549, expression NRF2 and its target protein HO-1 were significantly decreased in A549 with NRF2 knockout by western blot analysis. (C). The mRNA expression levels of NRF2 and its target genes were significantly decreased after the cell lines were knockout NRF2.(D) Colony-formation assay showed that the proliferation of A549 lung cancer cell lines depleted with NRF2 was significantly decreased.(E) The scratch wound-healing assay showed that the migration of A549 lung cancer cell lines depleted with NRF2 was slower at 0 h,24 h, and 48 h than that of A549 cell lines. Mean ± standard error of the mean (SEM) are reported (* P < 0.05; **, P < 0.01; ***, P < 0.001). [file 12964_2020_568_MOESM2_ESM.zip › supplymentary1.figure.tif]
